# Impact of COVID-19 on the mental health of men experiencing homelessness: A cross-sectional study in Osaka, Japan

**DOI:** 10.1371/journal.pone.0292377

**Published:** 2023-10-17

**Authors:** Masahiro Michinaka, Akira Sai, Taro Yamauchi

**Affiliations:** 1 Graduate School of Health Sciences, Hokkaido University, Sapporo, Hokkaido, Japan; 2 Global Station for Indigenous Studies and Cultural Diversity, Hokkaido University, Sapporo, Hokkaido, Japan; 3 Faculty of Health Sciences, Hokkaido University, Sapporo, Hokkaido, Japan; Uniformed Services University of the Health Sciences, UNITED STATES

## Abstract

The novel coronavirus infectious disease (COVID-19) pandemic has negatively impacted not only our physical health but also mental health, including increasing depressive and anxiety symptoms. In particular, socially and physically vulnerable populations, such as people experiencing homelessness (PEH), may be more likely to have their mental health worsened by the pandemic due to having more difficulty meeting basic human needs. Therefore, this study aims to assess the impact of COVID-19 on mental health of the homeless in Japan by evaluating depressive and anxiety symptoms and identifying the associated factors particularly, sociodemographic variables as age, employment status and the fear and perceived risk of COVID-19 infection. A cross-sectional interview survey among 158 PEH in Osaka Prefecture was conducted from April to May 2022. The survey included sociodemographic questions and history and perceived risk of infection with COVID-19. Depressive symptoms were measured using the nine-item Patient Health Questionnaire (PHQ-9) and anxiety symptoms using the seven-item Generalized Anxiety Disorder Scale (GAD-7), and the fear of COVID-19 using the seven-item Fear of New Coronavirus Scale (FCV-19S). In this study, the prevalence of depression (PHQ-9≥10) was 38.6%, anxiety disorder (GAD≥10) was 19.0%, and high fear of COVID-19 (FCV-19S≥19) was 28.5%. Univariate logistic regression analysis revealed that PEH in younger age groups (18–34 years), and with joblessness, higher perceived infection risk, and higher fear of COVID-19 were more likely to suffer from depression and anxiety (p<0.05). These results indicate that the younger PEHs with worsened economic conditions and therefore, feel threatened by COVID-19 the pandemic are at higher risk of mental health deterioration. More focused research and mental health services need to be provided to this population in the future.

## Introduction

In December 2019, a novel coronavirus infectious disease (COVID-19) caused a global pandemic that resulted in more than 6.5 million deaths [1]. Not only that, it brought about socioeconomic changes, changes in daily life, social isolation, fear of infection, and various other stressors that have negatively affected not only physical but also mental health such as depression and anxiety symptoms [2, 3]. The socioeconomic deterioration due to the pandemic increased the number of people who were unemployed or living in precarious conditions [4, 5]. People whose lives are unstable and socially disadvantaged are more likely to have deteriorated mental health due to chronically poor physical and mental health status caused by substance use and lack of access to social welfare [6].

People experiencing homelessness (PEH: homeless and unstably housed), who are socially and physically disadvantaged, may be more susceptible to the mental health effects of the pandemic [7]. PEH are at high risk of contracting a wide variety of infectious diseases due to their unsanitary living conditions and limited access to health services [8]. Some studies suggest that they have a higher rate of underlying disease and a higher risk of hospitalization and death due to the COVID-19 infection [9]. Factors that may contribute to the elevated fear of COVID-19 include having an underlying disease and concern about one’s own vulnerability to the infection [10, 11]. Therefore, it is plausible to predict that vulnerable populations, such as PEH, may experience increased fear and worsening mental health [12]. That said, on the other hand, previous experiences on disasters and traumatic events may act as a protective factor against adverse effects of the future events on one’s mental health [13], which needs to be examined.

Older PEH may be especially at risk of worsened mental status because of COVID-19. Past studies have reported that older adults have a higher fear of becoming infected with COVID-19 due to a higher risk of its severity and mortality due to the presence of underlying diseases [14]. Moreover, PEH in developed countries have been aging over the years, and Japan has been reported to have a relatively high percentage of older PEH [15]. Given the vulnerability of homelessness, as well as their advanced age, there is an urgent need to investigate the mental health of Japan’s population experiencing homelessness. It is critical to understand the impact of the pandemic on the mental health of PEH and to consider appropriate support to prevent exacerbated situations, such as suicide [16], due to the deterioration of mental health. To the best of our knowledge, this is the first study to explore mental health in PEH during the pandemic. This study aims to assess the impact on the mental health of PEH in Japan by studying the prevalence of depression and anxiety in this population during the COVID-19 pandemic, and identifying associated factors particularly, sociodemographic variables as age, employment status and the fear and perceived risk of COVID-19 infection.

## Materials and methods

### Study design and participants

The cross-sectional survey was conducted in Japan between April 24 and May 30, 2022. This period includes the period of the sixth wave of the pandemic, during which the number of cases of Omicron strains in Japan increased rapidly and a record number of cases were confirmed. Participants were recruited in person and interviewed by the first author at a soup kitchen and shelter by a homeless-supporting organization in Nishinari Ward, Osaka, Japan.

Participation criteria were age of 18 years or older and native Japanese speakers. Exclusion criteria were those who lived in their own houses without public assistance. Since most participants in this study were male (99%), the analysis was limited to males. In addition, responses that were found to be incomplete were excluded (response rate: 92%). Of the 177 respondents, 158 were included in the analysis through the above procedures. The interview was approximately 20 minutes long, and participants received a 500-yen (4 USD) gift card after the survey.

### Study instruments

The questionnaire had three sections as follows:

- Socio-demographic characteristics: Age, gender, place of residence in the last month (shelter, street, friend or acquaintance’s house, house on welfare, car, internet cafe, or cheap lodging house), education level (middle school graduate, high school graduate, or college graduate), and work status (day laborer or unemployed).
- Questions related to COVID-19: (1) History of infection with COVID-19: Participants were asked if they had ever been diagnosed as positive by a doctor in the hospital or experienced symptoms of COVID-19; (2) COVID-19 infection risk self-perception: Participants were asked if they were likely to be infected with COVID-19 infection. Hence, this was employed to assess one’s perceived risk of contracting COVID-19. Participants responded using a five-item Likert scale of (1 = very low, 2 = low, 3 = average, 4 = high, 5 = very high).
- Psychometric scales to assess depressive symptoms, anxiety symptoms, and fear of COVID-19:
  - The Patient Health Questionnaire-9 (PHQ-9) was used to assess depressive symptoms [17]. The scale is a nine-item brief screening test that assesses depressive symptoms. The severity of symptoms over the past two weeks was assessed. The nine items were scored based on a four-point Likert scale (0 = not at all, 1 = several days, 2 = more than half the days, 3 = nearly every day). Total scores range from zero to 27, with higher scores indicating more severe depressive symptoms. The severity of depressive symptoms was classified according to “minimal or none: 0–4 points,” “mild: 5–9 points,” “moderate: 10–14 points,” “moderately severe: 15–19 points,” and “severe: 20–27 points”. In this study, a cutoff value of ten points, often considered indicative of a likely depressive disorder, was used [18]. This tool has been validated in Japanese populations as a brief screening test for depression, and the Japanese version of the PHQ-9 was, hence, used in this study [19, 20].
  - The Generalized Anxiety Disorder-7 (GAD-7) questionnaire was used to assess anxiety symptoms. This scale is a seven-item screening test that was used to assesses the severity of symptoms over the previous two weeks [21]. The seven items were scored on a four-point Likert scale (0 = not at all, 1 = several days, 2 = more than half the days, 3 = nearly every day). The total score ranges from zero to 21, with higher scores indicating more severe anxiety symptoms. The severity of anxiety symptoms was classified according to “mild: 0–4 points,” “moderate: 5–9 points,” “moderately severe: 10–14 points,” and “severe: 15–21 points”. In this study, a cutoff value of ten points, often considered a likely anxiety disorder, was used [22]. This tool has been validated in Japanese populations as a brief screening measure for anxiety symptoms, and the Japanese version of the GAD-7 was, hence, used in this study [23].
  - Fear of COVID-19 scale (FCV-19S) was used to measure the fear of COVID-19. It consists of seven items to assess the levels of one’s general fear for COVID-19 (Sample item: “I am most afraid of coronavirus-19”) [24]. The participants responded to seven items on a five-point Likert scale (1 = strongly disagree, 2 = disagree, 3 = neither agree nor disagree, 4 = agree, 5 = strongly agree). The total score ranges from seven to 35, with higher scores indicating greater fear of COVID-19. In this study, the cutoff for high fear related to COVID-19 was 19 points or higher. This scale has been validated in Japanese, and the Japanese version of the FCV-19S was, hence, used in this study [25–27].

### Statistical analysis

Continuous variables were described as means and standard deviations in frequencies and percentages. The Shapiro-Wilk test was used to check the normality of the data for continuous variables. To evaluate the relationship between categorical variables (age, housing status, education level, day laborer, history of COVID-19 infection), logistic regression analysis was performed with the total scores of PHQ-9, GAD-7, and FCV-19S at the cut-offs (10 or above for PHQ-9 and GAD-7, and 19 or above for FCV-19s, respectively). The association of each variable was evaluated based on odds ratios (ORs) and 95% confidence intervals (CIs). Statistical analysis was performed using JMP version 16.2. The significance level was set at p < 0.05.

### Ethics statement

The study protocol and methods were reviewed and approved by the Ethics Review Committee of the Faculty of Health Sciences, Hokkaido University (No.21–68). All research participants were required to fill out an informed consent form after being briefed on the study.

## Results

### Participant characteristics

A summary of the participants’ characteristics is shown in Table 1. The sample used for statistical analysis consisted of 158 participants, with a mean age of 59.4 years (standard deviation: 11.5; range: 23–86). In the last month, 35.4% (n = 56) spent the most time in shelters, 15.2% (n = 24) on the street, 5.1% (n = 8) at a friend or acquaintance’s house, 29.7% (n = 47) in a house on welfare, and 14.6% (n = 23) in unspecified housing such as an internet cafe, car, or cheap lodging house. As for education, 9.5% (n = 15) completed college, which is the highest education level, whereas 41.8% (n = 66) completed middle school or lower and 48.7% (n = 77) completed highs school. 40.5% (n = 64) were employed as a day laborer and 14.6% (n = 23) were classified as having a history of COVID-19 infection. The mean perceived risk of contracting COVID-19 was 2.5 (SD ± 1.1), with most participants (n = 134, 85%) responding “very low,” “low,” or “average.”

**Table 1:** Participant characteristics.

	N/mean	%(SD)
**Age**	59.4	(11.5)
18–34 years	7	4.4
35–49 years	20	12.7
50–64 years	67	42.4
65–86 years	64	40.5
**Housed in the past 30days**		
Homeless shelter	56	35.4
Street/outdoors	24	15.2
Friend or acquaintance’s house	8	5.1
House on welfare	47	29.7
Unspecified residence ^a^	23	14.6
**Education level**		
Middle school or lower	66	41.8
High School	77	48.7
College	15	9.5
**Day laborer**		
Yes	64	40.5
No	94	59.5
**History of COVID-19 infection**		
Yes	23	14.6
No	135	85.4
**Risk perception** ^b^	2.5	(1.1)
Very low	34	21.5
Low	43	27.2
Average	57	36.1
Increased	17	10.8
High	7	4.4

^a^ Unspecified residence…PEH who do not live in a specific place, such as free and cheap lodging houses, cars, internet cafes.

^b^ Risk perception, Self-perceived risk of contracting COVID-19 (Min: 1, Max: 5)

### Mental health status

**Fig 1:**
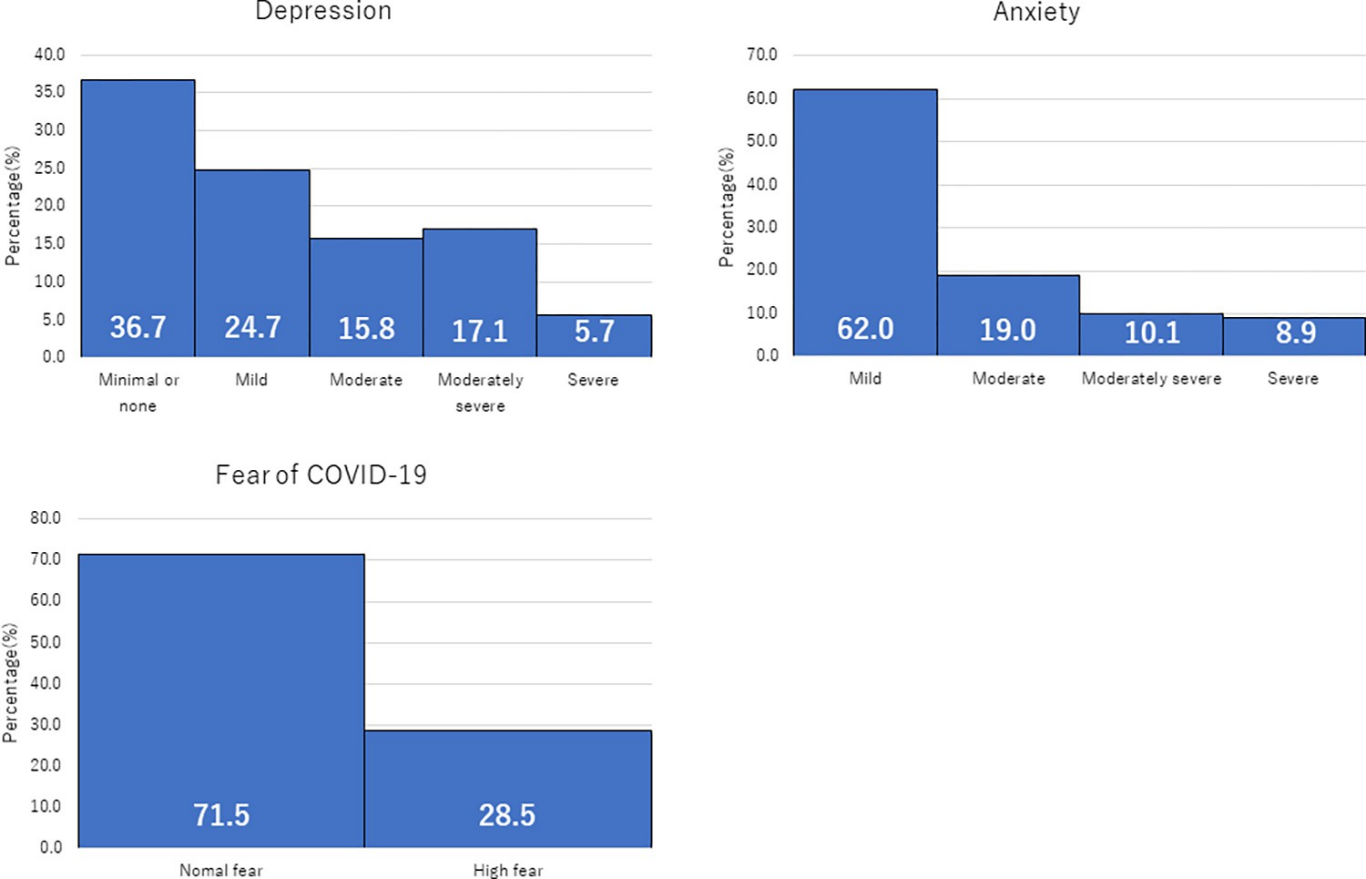
Prevalence of depression, anxiety and COVID-19 fear among homeless people.

The mean of the total PHQ-9 score was 8.22 (± 6.86) overall, with a median score of 7 (IQR: 3–14) (Table 2). The severity of depressive symptoms was 36.7% “minimal or none: 0–4”, 24.7% “mild: 5–9”, 15.8% “moderate: 10–14”, 17.1% “moderately severe: 15–19”, 5.7% “severe: 20–27”, and 38.6% (n = 61) had a total score of ten or more (depressive symptoms) (Fig 1). The mean of the total GAD-7 score was 4.70 (± 5.76) overall, with a median of 2 (IQR: 0–7) (Table 2). The severity of anxiety symptoms was 62.0% for “mild: 0–4”, 19.0% for “moderate: 5–9”, 10.1% for “moderately severe: 10–14”, 8.9% for “severe: 15–21”, and 19.0% (n = 30) for a total score of ten or more (with anxiety symptoms). The rates of these probable disorders are 38.6% (N = 61) for depressive symptoms and 19.0% (N = 30) for anxiety symptoms, respectively. The mean of the FCV-19S total score was 14.47 (± 7.50) overall, with a median score of 12.5 (IQR: 7.75–20) (Table 2). 28.5%(n = 45) of the respondents reported high levels of fear (total score of 19 or higher) (Fig 1).

**Table 2:** Correlation of PHQ-9, GAD-7, FCV-19s and age.

Variables	Mean (SD)	1	2	3	4	5
**1. PHQ-9**	8.22 (6.86)	-				
**2. GAD-7**	4.70 (5.76)	.77**	-			
**3. FCV-19S**	14.47 (7.50)	.29**	.40**	-		
**4. Age**	59.37 (11.54)	−.17*	−.23*	−.04	-	
**5.ssRisk perception**	2.49 (1.08)	.27**	.25*	.28**	−.18*	-

^1^ PHQ-9, Patient Health Questionnaire

^2^ GAD-7, Generalized Anxiety Disorder 7-item scale

^3^ FCV-19S, Fear of COVID-19 Scale

^4^ Age

^5^ Risk perception, Self-perceived risk of contracting COVID-19

*, significant at p<0.05

**, significant at p<0.001

Bivariate correlations were obtained to assess associations in key variables (Table 2). There was a significant positive correlation between FCV-19S, PHQ-9, and GAD-7. There was a trend toward weaker depressive (r = - 0.17, p < 0.05) and anxiety symptoms (r = - 0.23, p < 0.05) and infection risk perception (r = - 0.18, p < 0.05) with older age. In this regard, results also showed that younger participants were more likely to have increased symptoms. The higher the infection risk perception, the stronger the depressive symptoms (r = 0.27, p < 0.001), anxiety symptoms (r = 0.25, p < 0.05), and fear of COVID-19 (r = 0.28, p < 0.001) tended to be. No correlation was found between age and fear of COVID-19 (Table 2).

### Regression analysis of depression and anxiety among PEH

The associated factors of depressive and anxiety symptoms are shown in Table 3. Univariate logistic regression analysis showed that depressive symptoms tended to be stronger in PEH aged 18–34 compared to PEH aged 35–49 years [OR = 0.071, 95% CI = 0.007–0.729, p = 0.026], PEH aged 50–64 years (OR = 0.113, CI = 0.013–0.988 p = 0.049), PEH aged 65–86 (OR = 0.087, CI = 0.010–0.771; p = 0.028). Also, joblessness (OR = 2.155, CI = 1.092–4.251; p = 0.027), higher perceived infection risk (OR = 1.650, CI = 1.209–2.298; p < 0.001), and higher FCV-19S total score (OR = 1.063, CI = 1.017–1.111; p = 0.006) were significant predictors for depressive symptoms. Multivariate logistic regression analysis also supported the above age effects for anxiety, but not for depression (data not shown). Anxiety symptoms tended to be stronger in PEH aged 18–34 compared to PEH aged 30–49 (OR 0.071 =, CI = 0.009–0.547; p = 0.011), 50–64 (OR = 0.136, CI = 0.024–0.767; p = 0.024), and 65–86 (OR = 0. 034, CI = 0.001–0.221; p < 0.001). Higher perceived risk of infection (OR = 1.54, CI = 1.06–2.24; p = 0.004) and higher FCV-19S total score (OR = 1.109, CI = 1.052–1.169; p < 0.001) were also significantly predictive of anxiety symptoms.

**Table 3:** Regression analysis of depression and anxiety among PEH.

Variables	Depression		Anxiety		
	OR (95% CI)	p	OR (95% CI)		p
**Age**					
18–34 years	Reference		Reference		
35–49 years	0.071 (0.007–0.729)	0.026*	0.071 (0.009–0.547)		0.011*
50–64 years	0.113 (0.013–0.988)	0.049*	0.136 (0.024–0.767)		0.024*
65–86 years	0.087 (0.010–0.771)	0.028*	0.034 (0.001–0.221)		<0.001
**Housing status**					
Homeless shelter	Reference		Reference		
Street/outdoors	1.168 (0.432–3.159)	0.759	1.842 (0.520–6.520)		0.344
Friend or acquaintance’s house	0.278 (0.032–2.429)	0.247	-		-
House on welfare	1.714 (0.773–3.800)	0.185	2.676 (0.967–7.405)		0.058
Unspecified residence	1.498 (0.555–4.043)	0.425	1.944 (0.547–6.913)		0.304
**Education level**					
<High School	Reference		Reference		
High School	0.733 (0.373–1.441)	0.368	0.914 (0.385–2.169)		0.839
>High School	0.905 (0.289–2.836)	0.863	2.250 (0.650–7.794)		0.201
**Day laborer**					
Yes	Reference		Reference		
No	2.155 (1.092–4.251)	0.027*	1.459 (0.632–3.368)		0.376
**History of COVID-19 infection**					
Yes	Reference		Reference		
No	0.424 (0.173–1.040)	0.06	0.818 (0.277–2.414)		0.716
**Risk perception**	1.650 (1.209–2.298)	0.001**	1.538 (1.057–2.238)		0.022*
**Fear of COVID-19 score**	1.063 (1.017–1.111)	0.006**	1.109 (1.052–1.169)		<0.001

OR, Odds Ratio

Cl, confident interval

Risk perception, Self-perceived risk of contracting COVID-19

*** p < 0.05, ** < 0.01, *** < 0.001

## Discussion

This study investigated depressive and anxiety symptoms and their associated factors (e.g., age, housing types, socioeconomic status, perception of risk of contracting COVID-19, fear of COVID-19) in PEH to determine the psychological impact of the pandemic on them. Findings showed that during the pandemic, the prevalence of depression was 38.6%, while the prevalence of anxiety was 19.0% among the PEH in this study (Fig 1). Regression analysis revealed that younger age, joblessness, higher fear of COVID-19, and higher perceived risk of infection were contributing factors for the symptoms of anxiety disorders and depression (Table 3). On the other hand, 28.5% participants reported fear of COVID-19 (Fig 1), a percentage that was lower than that reported in previous surveys of the general population [28, 29]. One possible interpretation for this result is that PEH have already experienced disasters or traumatic events prior to the pandemic and therefore their resilience may have ultimately less affected the mental health of this population [13].

In this study, those with no job tended to show more depressive symptoms than those with a job (Table 3). This finding is consistent with previous studies showing that joblessness is associated with worse mental health during a pandemic crisis [30]. In this regard, it is plausible to think that the pandemic situation made it a jobless state more common among and thereby worsened their mental health. It has also been suggested that people in precarious situations, such as PEH, are more likely to face difficulties like unemployment and housing loss due to the pandemic [31]. Those who are unemployed or have lost income due to the pandemic’s effects, especially those whose future prospects, including occupational and economic prospects, are threatened by COVID-19, which may lead to a deterioration of their mental health [32, 33].

Surprisingly, younger age groups also showed higher depressive and anxiety symptoms than older age groups (Tables 2 and 3). While some studies have argued the role of age played in chronic illness, which ultimately leads to more prevalence of psychological distress among older participants [34, 35], several previous studies have reported that younger adults have higher depressive and anxiety symptoms than older adults [36–38]. One possibility is that it is due to financial problems caused by the pandemic or worries about future careers [39]. In addition, younger people who have been PEH for shorter periods of time may have experienced more elevated depressive and anxiety symptoms because of the new precarious situation of homelessness they faced because of the pandemic [40]. In this regard, the same explanation could apply not only to the pandemic but also various situations, where older PEH have more adaptability to the threats from both everyday life and unexpected disasters and traumatic events relative to younger PEH. Among the participants in this study, PEH who have experienced new lifestyle changes, such as unemployment, due to the pandemic are particularly likely to have experienced adverse mental health effects.

Higher fear of COVID-19 and higher perceived risk of infection contributed to worse depressive and anxiety symptoms (Tables 2 and 3). This supports the results of previous studies reporting that fear of infection with COVID-19 and risk perception have a negative impact on mental health [41]. As a socially vulnerable population, PEH may have difficulty obtaining adequate access to health services and sanitation facilities, potentially increasing their risk of infection. The high risk of infection is thought to increase fear, which in turn affects mental health. This suggests that fear of COVID-19 and perceived risk of infection are factors that worsen the mental health of the participants.

The rates of depressive and anxiety symptoms among the participants in this study were not substantially increased compared to those in the pre-pandemic PEH. Prior to the pandemic, one of the few studies of mental health status among PEH in Japan reported that 41.3% had depression and 15% had anxiety symptoms [42]. In addition, previous review articles have shown that the rates of depressive and anxiety symptoms in pre-pandemic PEH were 46.7% and 17.6%, respectively [43, 44], and, compared to the general population, the prevalence of depression was five to 14 times higher than in the general population [45]. Compared to studies conducted prior to the pandemic, there were no meaningful changes in the rates of depressive and anxiety symptoms among the participants in this study. This suggests that the impact of the pandemic on mental health may be smaller in PEH than in the general population, which also points to the need of further examining pre-post pandemic differences.

One possible reason for that pandemic impact on PEH’s mental health is smaller than that of general population that the social isolation experienced by the general population during the pandemic does not apply to PEH. Past studies have shown that restrictions such as social distancing and other measures to prevent the spread of infections can cause social isolation and negatively impact mental health, especially in the elderly [46, 47]. Nevertheless, the elderly (mean age 59.4 years), who comprised most of the participants in this study, had a lower risk of depression and anxiety disorders than the younger age groups (Tables 2 and 3). Since PEH have been shown to experience social isolation and loneliness even before the pandemic [48, 49], we expect a relatively small proportion of PEH to experience new social isolation after the pandemic. Thus, PEH who are thought to have been isolated to begin with are likely to have less negative impact on mental health in terms of aspects of social isolation.

In addition, the resilience of the older PEH may have been able to cope with the changes in their daily lives caused by the pandemic. Compared to young people, older adults may have improved resilience because they have experienced various major life events in the past, such as infectious disease pandemics and financial crises [50]. A study in the general population in Japan similarly suggests that the elderly are better able to cope with pandemic stress than younger people [51]. In addition, PEH may face far more significant daily problems and greater threats than the COVID-19 outbreak because of their precarious homelessness [52]. Based on these findings, it is possible that the elderly PEH may be more experienced in coping with the changes in their daily life caused by COVID-19, thus reducing the psychological impact of the pandemic.

Another possible explanation for the smaller mental health impact of the pandemic on PEH is the low fear of COVID-19 among the PEH in this study. Only 28.5% of the participants (mean score 14.47) reported a high fear of COVID-19 (Fig 1). These results were lower than those reported in a study using a similar questionnaire in a general population of Japanese university students, hospital nurses, and pregnant women [53]. Although infection risk perception was associated with the fear of COVID-19 (Table 2), we believe the mean score for infection risk perception was not high (mean 2.5), leading to a low fear of infection. This may be because the PEH experience social isolation on a daily basis and, therefore, have fewer opportunities for social interaction and contact with others that increases their infection risk perception. In addition, PEH with less social interaction than before the pandemic may be less likely to have factors that increase COVID-19 fear, such as infection of family members, presence of an infected person close by [54, 55], and stigma from others due to their own infection [56], and other factors that increase COVID-19 fears. Other than the above, it is also possible that living outside and trying hard to make ends meet may explain less fear of COVID-19, which need to be further examined.

There are several limitations to this study. First, because this is a cross-sectional study, it is difficult to assert the impact of the COVID-19 pandemic on depressive and anxiety symptoms among PEH. Longitudinal studies are needed to better understand the impact of PEH on mental health status. Second, only men were included in the analysis. Since gender has been reported to be an influencing factor in mental status and COVID-19 fear [57], the findings of this study should be compared with the general population with caution. In this regard, it is important to shed light on female PEH to have a more comprehensive understanding of this population. Despite these limitations, this study was conducted in a large-scale face-to-face interview survey of PEH who were difficult to contact due to the pandemic disaster. The results of this survey will provide important insights for the future.

## Conclusions

During the pandemic, the prevalence of depression and anxiety among 158 PEH in Japan was 38.6% and 19.0%, respectively. Also, being younger, being unemployed, having a higher fear of COVID-19, and having a higher perceived risk of infection were factors that worsened mental health. These results indicate that PEH who are forced to make lifestyle changes due to COVID-19, such as unemployed and young PEH, may be negatively affected in their mental health, which points to that this population is more likely to be at risk for worsening compared to others. It is also worthwhile to note that mental health status during the pandemic was not significantly different from that of pre-pandemic among PEH in Japan, which would be helpful for the PEH supporting scholars and organizations to further investigate how country and region specific social and cultural factors contribute to this phenomenon.

## Supporting information

S1 Data (XLSX)
